# Using information from network meta-analyses to optimize the power and sample allocation of a subsequent trial with a new treatment

**DOI:** 10.1186/s12874-022-01792-6

**Published:** 2022-11-22

**Authors:** Dapeng Hu, Chong Wang, Fangshu Ye, Annette M. O’Connor

**Affiliations:** 1 Department of Statistics, College of Liberal Arts and Sciences, Iowa State University, Ames, IA United States of America; 2 Department of Veterinary Diagnostic and Production Animal Medicine, College of Veterinary Medicine, Iowa State University, Ames, IA United States of America; 3 Department of Large Animal Clinical Sciences, College of Veterinary Medicine, Michigan State University, East Lansing, MI United States of America

**Keywords:** Network meta-analysis, Sample size, Clinical trial design, Evidence synthesis

## Abstract

**Background:**

A critical step in trial design is determining the sample size and sample allocation to ensure the proposed study has sufficient power to test the hypothesis of interest: superiority, equivalence, or non-inferiority. When data are available from prior trials and leveraged with the new trial to answer the scientific questions, the value of society’s investment in prior research is increased. When prior information is available, the trial design including the sample size and allocation should be adapted accordingly, yet the current approach to trial design does not utilize such information. Ensuring we maximize the value of prior research is essential as there are always constraints on resources, either physical or financial, and designing a trial with adequate power can be a challenge.

**Methods:**

We propose an approach to increasing the power of a new trial by incorporating evidence from a network meta-analysis into the new trial design and analysis. We illustrate the methodology through an example network meta-analysis, where the goal is to identify the optimal allocation ratio for the new three-arm trial, which involves the reference treatment, the new treatment, and the negative control. The primary goal of the new trial is to show that the new treatment is non-inferior to the reference treatment. It may also be of interest to know if the new treatment is superior to the negative control. We propose an optimal treatment allocation strategy which is derived from minimizing the standard error of the log odds ratio estimate of the comparison of interest. We conducted a simulation study to assess the proposed methods to design a new trial while borrowing information from the existing network meta-analysis and compare it to even allocation methods.

**Results:**

Using mathematical derivation and simulations, we document that our proposed approach can borrow information from a network meta-analysis to modify the treatment allocation ratio and increase the power of the new trial given a fixed total sample size or to reduce the total sample size needed to reach a desired power.

**Conclusions:**

When prior evidence about the hypotheses of interest is available, the traditional equal allocation strategy is not the most powerful approach anymore. Our proposed methodology can improve the power of trial design, reduce the cost of trials, and maximize the utility of prior investments in research.

## Background

Randomized controlled trials (RCTs) are the gold standard approach to evaluating new treatments. However, a single RCT is unlikely to be conclusive unless it is very large and multi-centered. Pairwise meta-analysis and network meta-analysis (NMA) are tools used for summarizing and combining the existing evidence from a network of RCTs. The advantage of meta-analysis is that it incorporates heterogeneity within and between trials and therefore gives a pooled estimate of comparative efficacy using all the information available. When data are available from prior trials in a meta-analysis, it has been proposed that such prior information can be used in designing new trials and then the new trial results could immediately be incorporated into a meta-analysis to obtain an updated pooled estimate of comparative efficacy [1–7]. An obvious advantage of leveraging evidence from the network is that it could reduce the required sample size or increase the power of the new trial.

Methods to plan a future trial based on the trial network from a pairwise meta-analysis have been proposed [1–3]. The methods have been extended to NMAs [4–6]. Incorporating evidence from an NMA into new trial design offers unique advantages over pairwise meta-analysis because of the potential to leverage both direct and indirect evidence available from the network. Planning future studies to reduce the existing network’s irregularity has also been discussed [8]. The approaches to trial design informed by NMA published to date have often focused on designing either multiple trials or a single trial that will achieve a desired power or estimation precision regarding existing treatments in the entire network [4–6, 8].

There does appear to be interest in the idea of leveraging prior research to design trials. In a survey on 76 researchers regarding evidence-based planning of future trials [9], two thirds of participants were agreeable to considering synthesizing existing evidence to design a future trial. There has been, however, little published work utilizing such methodologies in designing new trials to date.

A common goal of conducting a new clinical trial is to evaluate a new treatment against existing reference treatments and/or negative control. Interestingly, the previously published approaches have not addressed using the existing network to design a new trial when one of the treatments of interest is not currently included in the existing network. Here we propose an approach to using information from the existing network meta-analysis to plan a trial with a new treatment not included in the existing network. The research question is how to optimize the power or total sample size of the future trial which includes a new treatment of interest. Gains in power or reductions in required total sample size are achieved over the traditional stand-alone power calculations by leveraging information from the network of evidence and evaluating optimal ways to allocate study subjects to the treatment groups. The methodological results are accomplished by an R shiny [10] application that allows end-users to upload a prior network and determine the most powerful treatment allocation approach for a future three-arm trial with a novel treatment.

## Methods

We illustrate our proposed approach using a three-arm clinical trial where the goal is to test non-inferiority between a new treatment and a reference treatment. This was motivated by an application to the use of antibiotics to treat bovine respiratory disease (BRD). The question of non-inferiority was chosen because it is often more meaningful and realistic than asking if the new treatment is superior to current therapy. The new product might be equal in efficacy but cheaper or have other favorable attributes which would still motivate switching treatments. However, non-inferiority clinical trials are often underpowered or require large sample sizes due to the small comparative effect size between the new treatment and the reference treatment. The assessment of non-inferiority can be conducted in a two-arm between the referent and new treatments. However, if there is a concern that the reference treatment (active treatment) might not be superior to the placebo (negative control) due to the absence of reliable historical data or disease etiology, then a negative control group should be included [11]. Thus it is common practice that at least three arms, the new treatment, the reference treatment, and the negative control, are included in such trials to study comparative effectiveness of treatments [12–14].

The organization of the sections is as follows. In "Comparative effect size estimation in a traditional three-arm trial" section, we review traditional comparative effect size estimation and its variation in a three-arm trial which forms the basis for sample size and power calculations. Comparative effect size estimation of the new trial with existing fixed effects NMA model section provides the framework and formula used for the approach to analyse a three-arm trial with a fixed effects network meta-analysis. Optimal sample allocation with a fixed total sample size section discusses allocation of study subjects to the treatment arm with a fixed total sample size for minimization of the variance of the effect estimation with an existing network. Design the new trial with the existing network to achieve a desired power section explores the approach to calculating the required sample sizes for a new trial with the existing network to achieve a desired power.

### Comparative effect size estimation in a traditional three-arm trial

Suppose we need to conduct a new three-arm trial comparing treatment A, B and Z on a binary outcome, where A and B are treatments that already exist in the network (for example, A is negative control and B is reference treatment) and Z is a new treatment. Here we discuss a standard three-arm trial design with simple random sampling from the study population and random allocation to treatment groups. The study population should be chosen to be consistent with the studies in the existing NMA. Suppose the total sample size available for the new trial is fixed, denoted by *n*, and we are most interested in the comparison of Z to B. Let the number of samples assigned to treatment A, B, and Z in the new trial be $n_A$, $n_B$, and $n_Z$, respectively, under the condition $n_A + n_B + n_Z = n$. Let $r_i$ denote the number of events among the subjects that have taken the *i*th treatment, $i \in \{A, B, Z\}$. Then the number of events follows a binomial model,

$$\begin{aligned} r_i \sim \text {binomial}(n_i, \pi _i) , \end{aligned}$$

where $\pi _i$ is the probability of event for treatment *i*. Utilizing a logistic model,

1 $$\begin{aligned} \text {log}(\frac{\pi _i}{1-\pi _i}) = \beta _1 + \beta _2 I_{(i = B)} + \beta _3 I_{(i = Z)} , \end{aligned}$$

the maximum likelihood estimation approach can be used to estimate the coefficients $\varvec{\beta }= (\beta _1, \beta _2, \beta _3)'$ and obtain the Hessian matrix. In Eq. 1, $\beta _1$ is the log odds of the probability of events in subjects that have taken treatment A; $\beta _2$ and $\beta _3$ are the log odds ratios of treatment B over A and Z over A, respectively. Compared to the general notation of treatment effect in NMA literature, where $\mu _{XY}$ denotes the log odds ratio of treatment *Y* over treatment *X* for any two treatments *Y* and *X*, we have the equivalences $\mu _{BA}=\beta _2$, $\mu _{ZA}=\beta _3$ and $\mu _{BZ}=\beta _3 - \beta _2$. The estimation variance of the comparative effect size of treatment Z to B, $\mu _{BZ}$, is

2 $$\begin{aligned} \text {Var}(\hat{\mu }_{BZ}) = \text {Var}(\hat{\beta _3} - \hat{\beta _2}) = \frac{1}{n_B p_B (1-p_B)} + \frac{1}{n_Z p_Z (1-p_Z)}, \end{aligned}$$

where $$p_i = r_i/n_i,$$ $$i \in \{A, B, Z\}$$.

Under the condition that $n_B + n_Z$ is fixed and $p_B = p_Z$, it can be shown by the inequality of arithmetic and geometric means that $\text {Var}(\hat{\mu }_{BZ})$ is minimized when $n_B = n_Z$. Hence, without evidence from the existing network, an equal allocation approach will minimize the variance if B and Z have the same efficacy.

### Comparative effect size estimation of the new trial with existing fixed effects NMA model

Consider a network of *T* treatments involving treatment A and B with *J* studies, and $n_j$ arms in the *j*th study, $j = 1,...,J$. Note that not all studies compare all treatments. For example, in a network with three treatments, studies comparing only pairs of treatments can also be included. There are ${T \atopwithdelims ()2}$ possible pairwise comparisons in the network, each associated with a comparative treatment effect parameter. We denote the set of comparative treatment effect parameters by $\varvec{\mu }_f$. Let $\varvec{\mu }_b = (\mu _{AB}, \mu _{AC},\mu _{AD}, ...)'$ be a sub-vector of $\varvec{\mu }_f$ of length $T-1$ that involves the comparative effect parameters of all treatments to a baseline. Here the baseline is assumed to be treatment A which is the negative control, however the choice of baseline treatment does not impact the results of NMA [15]. The vector $\varvec{\mu }_b$ is the vector of basic parameters. We assume that the transitivity and consistency assumptions are met for a network meta-analysis [16–18]. In particular, under the consistency assumption, the vector $\varvec{\mu }_f$ representing the comparative treatment effects between all pairwise comparisons is a linear combinations of $\varvec{\mu }_b$. For example, $\mu _{BC} = \mu _{AC} - \mu _{AB}$.

In a fixed effects NMA we assume that there is one comparative effect size underlying the trials for each comparison. It follows that all of the differences in the observed comparative effect sizes of a pairwise comparison are due to random variation (sampling error) [19]. Let $\varvec{y}_j$ denote the observed comparative effect size for the *j*th study, $\varvec{y}_j = (y_{j,1},..., y_{j,n_j-1})'$, and $\varvec{y} = (\varvec{y}_1', ... ,\varvec{y}_J')'$. Let $\varvec{\mu }_j$ be the vector of the comparative effect sizes of study *j*, $\varvec{\mu }_j = (\mu _{j,1},..., \mu _{j,n_j-1})'$, and $\varvec{\mu } = (\varvec{\mu }_1',...,\varvec{\mu }_J')'$. Then we have

$$\begin{aligned} \varvec{y}_j = \varvec{\mu }_j + \varvec{\epsilon }_j, \quad j = 1,...,J, \end{aligned}$$

where $\varvec{\epsilon }_j$ represents the vector of errors of study *j*. $\varvec{\epsilon }_j$ is assumed to be normally distributed and independent across studies and its covariance is cov($\varvec{\epsilon }_j$) = $\varvec{S}_j$. $\varvec{S}_j$ is a matrix of size $(n_j - 1) \times (n_j - 1)$ and is a scalar if study *j* only has two arms. The distribution of $\varvec{y}$ is $\textrm{MVN}\left( \varvec{\mu }, \varvec{S}\right) ,$ where MVN stands for multivariate normal distribution; $\varvec{S}$ is a block diagonal matrix with each block $\varvec{S}_j, j = 1,...,J$. Since $\varvec{\mu }$ is also a linear combination of $\varvec{\mu }_b$, it can be written as $\varvec{\mu } = \varvec{X}\varvec{\mu }_b$, where $\varvec{X}$ is the design matrix of size $\sum _{j=1}^{J} n_j \times (T-1)$. Each row of $\varvec{X}$ corresponds to one study specific comparison and the columns represent the basic parameters, and 1, 0, and -1 are the possible values in the design matrix. If one row of $\varvec{X}$ only has one element of 1 and other elements are 0, then this study specific comparison is a basic comparison. If 1 and -1 occur in one row, then the comparative effect parameter of the corresponding comparison is a functional parameter. The distribution of $\varvec{y}$ is then $\textrm{MVN}\left( \varvec{X}\varvec{\mu }_b, \varvec{S}\right) .$ The maximum likelihood estimate of $\varvec{\mu }_b$ and its variance are

3 $$\begin{aligned}{} & {} \hat{\varvec{\mu }}_b = (\varvec{X}' \varvec{S}^{-1} \varvec{X})^{-1} \varvec{X}' \varvec{S}^{-1} \varvec{y}, \nonumber \\{} & {} \text {Var}{(\hat{\varvec{\mu }}_b)} = (\varvec{X}' \varvec{S}^{-1} \varvec{X})^{-1}. \end{aligned}$$

Now consider designing the new trial comparing A, B and Z with the existing network. Since treatment A and B are included in the network, we can borrow some information from the existing network to improve the precision of estimate of the effect of treatment Z compared to B. Evidence can be borrowed from the network for comparing Z and B since the indirect comparison Z-A-B can be constructed. By borrowing information from the indirect comparison Z-A-B, the standard error of the estimate of the comparative effect size of Z to B could decrease. Let the variance of the estimate of the comparative effect size of B to A calculated from the existing network be $\sigma _{AB,exg}^{2}$, where the subscript *exg* represents the existing network. Let the within-study variance of the three comparisons in the new trial be $\sigma ^{2}_{AB}, \sigma ^{2}_{BZ}, \sigma ^{2}_{AZ}$. Without loss of generality, A is assumed to be the baseline treatment in the NMA. Then, the new trial is added to the existing network to become an updated network. The variance-covariance matrix of the new trial, denoted by $\varvec{S}_{new}$, the variance-covariance matrix of the updated network, $\varvec{S}^{*}$, and the updated design matrix, $\varvec{X}^{*}$ are

4 $$\begin{aligned} \varvec{S}_{new} = \left[ \begin{array}{cc} \sigma ^{2}_{AB} &{} \frac{\sigma ^{2}_{AB} + \sigma ^{2}_{AZ} - \sigma ^{2}_{BZ}}{2}\\ \frac{\sigma ^{2}_{AB} + \sigma ^{2}_{AZ} - \sigma ^{2}_{BZ}}{2} &{} \sigma ^{2}_{AZ} \end{array}\right] , \quad \varvec{S}^{*} = \left[ \begin{array}{cc} \varvec{S} &{} \varvec{0}\\ \varvec{0} &{} \varvec{S}_{new} \end{array}\right] , \quad \varvec{X}_* = \left[ \begin{array}{cc} \varvec{X} &{} \varvec{0} \\ \varvec{X}_{new} &{} \varvec{Q}_{new} \end{array}\right] , \end{aligned}$$

where $\varvec{X}_{new}$ and $\varvec{Q}_{new}$ are separate parts of the design matrix of the new trial [20]. The columns in $\varvec{X}_{new}$ are for the previous basic parameters while $\varvec{Q}_{new}$ is a $2 \times 1$ vector of design for the new basic parameter $\mu _{AZ}$. The estimate of comparative effect size of Z to B is $\hat{\mu }_{BZ} = \hat{\mu }_{AZ} - \hat{\mu }_{AB}$. From the formula 3 and 4, the variance of $\hat{\mu }_{BZ}$ is then given by

5 $$\begin{aligned} \begin{aligned} \text {Var}(\hat{\mu }_{BZ})=&\frac{1}{n_B p_B (1-p_B)} + \frac{1}{n_Z p_Z (1-p_Z)} \\&- \frac{1}{[n_B p_B (1-p_B)]^{2} (\sigma ^{2}_{AB, exg}+\frac{1}{n_A p_A (1-p_A)} + \frac{1}{n_B p_B (1-p_B)})}. \end{aligned} \end{aligned}$$

By comparing Eq. 2 with Eq. 5, we can find that under the even allocation condition where $n_A = n_B = n_Z$, $\text {Var}(\hat{\mu }_{BZ})$ in Eq. 5 is less than that in Eq. 2 because of the third term in Eq. 5. This amount of reduction in variance is the contribution of borrowing information from the existing network.

### Optimal sample allocation with a fixed total sample size

In "Comparative effect size estimation of the new trial with existing fixed effects NMA model" section, we showed that under the even allocation condition, the variance of the comparative effect size of interest can be reduced. Whether the even allocation condition is the optimal sample allocation strategy in terms of minimizing the variance remains unknown. The variance formula is a function of the allocation: $\text {Var}(\hat{\mu }_{BZ}) = f(n_A, n_B, n_Z)$. Based on its derivation, the allocation strategy for the new trial is an optimization problem given by:

6 $$\begin{aligned} \begin{array}{ll} &{}\text {minimize } f(n_A, n_B, n_Z) = \\ &{}\frac{1}{n_B p_B (1-p_B)} + \frac{1}{n_Z p_Z (1-p_Z)} - \frac{1}{[n_B p_B (1-p_B)]^{2} (\sigma ^{2}_{AB, exg}+\frac{1}{n_A p_A (1-p_A)} + \frac{1}{n_B p_B (1-p_B)})} \end{array} \end{aligned}$$

$$\begin{aligned} \begin{array}{l} \text {s.t }\ n_A + n_B + n_C = n\\ n_A, n_B \ \text { and } n_C\ge 10\\ n_A, n_B \ \text { and } n_C \text { are positive integers}. \end{array} \end{aligned}$$

We set the constraint on the minimum number of samples for each treatment to be 10 to ensure the statistical inference on the new trial is based on a reasonable number of subject in each group. Without this constraint samples size of 1 is a possible solution mathematically, which is not practically feasible though, especially when the outcome is binary. The constraint value 10 can be changed to other appropriate numbers, such as 3 or 5, depending on the exact trial study scenario. Closed form solutions do not exist for minimizing this function. Therefore, nonlinear optimization methods can be used for obtaining the optimal allocation. In particular, we utilize the “differential evolution optimization” [21]. Although this optimization method is used over a continuous space, the integer solution can be obtained by enumerating all possible integers around the global solution.

### Design the new trial with the existing network to achieve a desired power

Optimal sample size allocation can be used to minimize the standard error of the estimates of the comparison of interest to maximize the power given a fixed total sample size; and it can also be used to minimize the total sample size given a desired power. We illustrate this with a non-inferiority example. If the research question is about non-inferiority, then the hypotheses are

$$\begin{aligned} H_0: \mu _{BZ} >= M, \quad H_1: \mu _{BZ} < M, \end{aligned}$$

where M is the non-inferiority margin which is specified in advance. The power of testing the non-inferiority hypothesis is given by

7 $$\begin{aligned} \text {Power}=\Phi (-\frac{\mu _{BZ} - M}{\sqrt{\text {Var}(\hat{\mu }_{BZ}})}-Z_{\alpha }), \end{aligned}$$

where $\Phi (\cdot )$ is the standard normal cumulative distribution function; $Z_{\alpha /2}$ is the upper $\alpha /2$th quantile of the standard normal distribution.

If the research question is about detecting a difference, then let the null hypothesis $H_0$ and alternative $H_1$ be $\mu _{AZ} = 0$ and $\mu _{AZ} \ne 0$, respectively. Once the minimized variance of $\hat{\mu }_{AZ}$ is obtained, the power is given by

8 $$\begin{aligned} \text {Power}=\Phi (\frac{\mu _{AZ}}{\sqrt{\text {Var}(\hat{\mu }_{AZ}})}-Z_{\alpha /2})+\Phi (-\frac{\mu _{AZ}}{\sqrt{\text {Var}(\hat{\mu }_{AZ}})}-Z_{\alpha /2}). \end{aligned}$$

Note that in a three-arm trial it is not possible to maximize the testing power of two comparisons simultaneously. With a predefined power, one can calculate the corresponding standard error and then numerically back-calculate the sample size and its allocation.

### R shiny application

The formulae above outline the basis for the sample size and/or power calculation. Moreover, to facilitate using these formulae we also provide an R shiny app (https://amoconnor.shinyapps.io/NMA-three-arms-sample-size/) that enables users to implement the proposed method. The instructions for data formatting and requirements can be found in the R-shiny app. Users can upload a NMA dataset and select the control and referent treatments to be included in the new trial. Users can also specify the comparison of interest, type of testing (e.g. superiority, non-inferiority or bioequivalence), and trial restriction (e.g. fixed total sample size or a desired power). Based on the data and the settings, this application can automatically calculate the optimal sample size allocation.

## Application and Simulation

Here we discuss an application to use of antibiotics for the treatment of bovine respiratory disease (BRD) and conduct a simulation study to assess the performance of the proposed methods to design a new trial while borrowing information from the existing NMA and compare it to traditional methods.

### Application

**Fig. 1:**
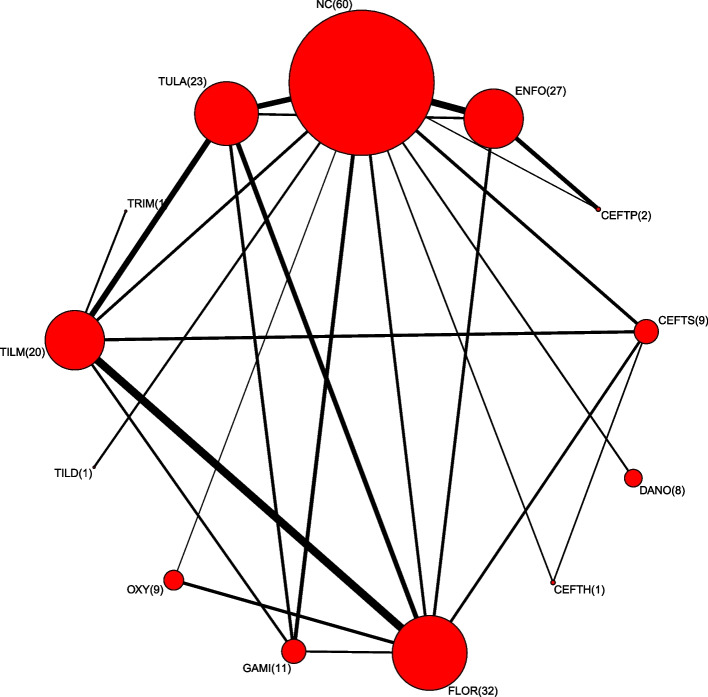
Bovine respiratory disease (BRD) network consisting of 13 antibiotics treatments

In this section, the dataset comes from a previously published network meta-analysis and consists of 98 trials, each comparing a subset of 13 treatments of antibiotics for BRD [22, 23]. Most trials contain two arms, except for eight three-arm trials. The outcome is the dichotomous disease status, and the log odds ratio of the outcome is used to compare treatments, where a negative comparative effect size of treatment A to B indicates that A is better. A network plot is shown in Fig. 1. Note that most of the trials in the network involve comparisons with negative control.

In BRD, three-arm designs are common because BRD is a disease syndrome with complex etiology that can involve bacteria and virus agents [24–26]. The causes are not established in individual trials or animals. Therefore, it is feasible that in any particular trial population, the disease might be viral in origin and resistant to antibiotic treatment. In such a study population with viral-based BRD, we would expect recovery to be the same in both treatment groups and the negative control, thus the inclusion of a negative control would help to document that no treatment was effective. Due to this situation, even historical data is unlikely to provide a sufficient rationale for the omission of a negative control for non-inferiority studies because BRD is unique in each population.

**Fig. 2:**
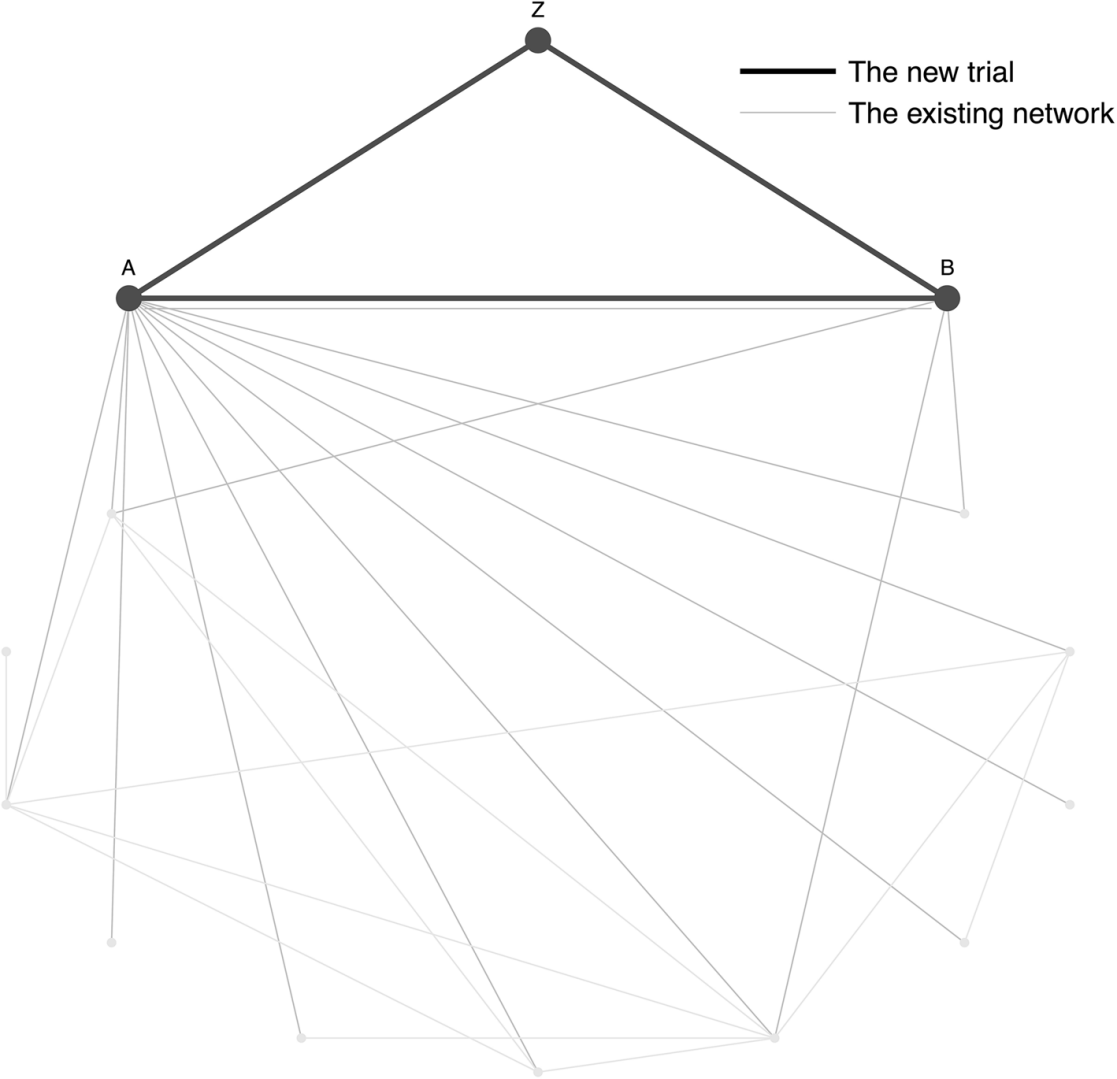
A new three-arm trial with a new treatment Z and two existing treatments

Suppose, researchers of BRD would like to conduct a new three-arm trial with a new treatment Z and two treatments existing in the current network, as illustrated in Fig. 2. In the following subsections, we will discuss several scenarios in which the new three-arm trial is going to be conducted. For each scenario, our proposed method is utilized first to calculate the optimal sample size allocation. The new trial data is then generated and analysed following the procedure described in "Simulation data generation and analysis procedure" section using both the proposed optimal allocation and the traditional even allocation. Trial scenario 1: testing non-inferiority section discusses application and performance of our proposed methodology using example trial scenarios for testing non-inferiority. Last, an application of our proposed method in a trial scenario for testing superiority is discussed in "Trial scenario 2: testing superiority" section. As discussed previously, the network meta analysis in this section is performed using a fixed effect contrast-based model.

### Simulation data generation and analysis procedure

A summary of the procedure to generate and analyze the new trial data is described as follows:

1. From a network meta-analysis of the existing network, the expected probability of the outcome in treatment A and B, respectively $\pi _A$ and $\pi _B$, can be estimated. The expected probability of the outcome in treatment Z, $\pi _Z$, can be obtained either from a preliminary study or from an expert.
2. Data representing the new study is generated by simulating $r_i$ samples from a binomial distribution Binom($n_i,p_i$), $i \in \{A, B, Z\}$, where $r_i$ is the number of outcome events in treatment *i*.
3. The simulated data can be analysed by a generalized linear model and the point estimate and standard error of the comparative effect size (log odds ratio) of treatment Z to B can be obtained from the result.
4. The result of the analysis of the new study is then added to the existing network, which is then re-analysed.
5. The hypothesis test on which decisions are to be based for the comparative effect size of treatment Z to B is then considered and whether the null is rejected at a specified significance level ($\alpha = 0.05$ in this paper) is noted.
6. Steps 2-5 are repeated (10,000 in this paper) and the outcome of each hypothesis test (step 5) is recorded. The results are summarized and reported as power or type I error rate for hypothesis testing.

The criteria for superiority of a treatment to the negative control is that the upper limit of the 95% one-sided confidence interval of the log odds ratio of the treatment to the negative control is lower than zero. The criteria for non-inferiority of the new treatment to the reference treatment is that the upper limit of the 95% one-sided confidence interval (the lower limit is $-\infty$ because the non-inferiority test is one-sided) of the log odds ratio should be lower than a predefined non-inferiority margin *M* (as in Eq. 7) which is set to be 0.2 in the application of testing non-inferiority.

In "Trial scenario 1: testing non-inferiority" section, we illustrate applications of our methodology using an example of assessing non-inferiority. In the first application, we assume the total sample size is fixed, and in the second application we assume a desired power is needed. The new three-arm trial has a negative control (NC), enrofloxacin (ENFO) and the new treatment Z. In this simulation, we assume that Z is non-inferior to ENFO and it has exactly the same probability of the outcome, in this case recurrence of disease, as ENFO. The probability of the outcome in the NC group ($p_{NC}$) is 0.681 and is estimated by $\sum _j r_j/ \sum _j n_j$, where *j* is the *j*th trial that includes NC. The probability of the outcome in the ENFO group ($p_{ENFO}$) is calculated from the log odds ratio of NC to ENFO, denoted by $LOR_{ENFO,NC}$, which is obtained from the fixed network meta-analysis model analysis. That is $p_{ENFO} = {p_{NC}\cdot e^{-LOR_{ENFO,NC}}}/({p_{NC}\cdot e^{-LOR_{ENFO,NC}} + 1 - p_{NC}}) = 0.2229$, where $LOR_{ENFO,NC} = 2.007$. Another quantity obtained from the fixed network meta-analysis model is the standard error of $LOR_{ENFO,NC}$ which plays a role in determining the optimal sample allocation.

In "Trial scenario 2: testing superiority" section, we show that the methodology can be applied to superiority trials.

### Trial scenario 1: testing non-inferiority

#### Fixed total sample size

In this example, the total sample size available for all three treatments are 2400, 3000, or 3600. Large samples sizes are not unexpected for a test of equivalence for a highly effective treatment. For each total sample size limit we compare three scenarios: even allocation of study subjects without the existing network, even allocation of study subjects with the existing network, and optimal allocation of study subjects with the existing network. Under the null hypothesis when testing non-inferiority, the new treatment Z is classified as non-inferior (reject the null hypothesis) to the ENFO in terms of efficacy if the upper boundary of the 95% confidence interval of the estimates of the log odds ratio of Z to ENFO is less than 0.2. For example if the upper limit of 95% confidence interval is 0.1 we would reject the null and conclude Z is non-inferior to ENFO. The null hypothesis of testing non-inferiority is that Z is inferior to ENFO. To assess the type I error rate, we set the comparative effect size of Z to ENFO to 0.2 which is equal to the predefined non-inferiority margin and then back-calculate the probability of retreatment of Z which is 0.2613. The difference is probability of retreatment of Z and ENFO indicates that Z is inferior to ENFO. The type I error rate of testing non-inferiority from the simulations are reported in Table 1. The type I error rate is well controlled for all scenarios.

**Table 1:** Type I error rate of testing non-inferiority for different fixed sample sizes under different sample allocation scenarios. $p_{NC}$ is the probability of retreatment after beef cattle being treated with negative control. ENFO is enrofloxacin; Z is the new treatment. The log odds ratio of Z to ENFO is 0.2 which indicates Z is inferior to ENFO. Sample allocation is the number of samples assigned to each treatment. For example, (87, 1140, 1173) means the number of samples assigned to NC, ENFO, and Z are 87, 1140, and 1173

total sample size	$p_{NC}$	$p_{ENFO}$	$p_{Z}$	sample allocation (NC, ENRO, Z)	with existing network	non-inferiority type I error
2400	0.681	0.2229	0.2613	(800, 800, 800)	No	4.24%
2400	0.681	0.2229	0.2613	(800, 800, 800)	Yes	3.83%
2400	0.681	0.2229	0.2613	(87, 1140, 1173)	Yes	4.02%
3000	0.681	0.2229	0.2613	(1000, 1000, 1000)	No	3.87%
3000	0.681	0.2229	0.2613	(1000, 1000, 1000)	Yes	3.32%
3000	0.681	0.2229	0.2613	(87, 1448, 1465)	Yes	3.91%
3600	0.681	0.2229	0.2613	(1200, 1200, 1200)	No	3.85%
3600	0.681	0.2229	0.2613	(1200, 1200, 1200)	Yes	3.28%
3600	0.681	0.2229	0.2613	(87, 1757, 1756)	Yes	3.66%

To assess the power of a study with a fixed sample size, the comparative effect size of Z to ENFO is set to 0 which means these two treatments have the same probability of retreatment , 0.2229 to ensure Z is indeed non-inferior to ENFO. The power analysis results are shown in Table 2. In the even allocation strategy, the power of the non-inferiority comparison between Z and ENFO in the network meta-analysis with the new trial is about 8% higher than that in the single trial analysis for all sample sizes listed. It means that the indirect evidence from the comparison between NC and ENFO in the existing network, as a part of the indirect evidence, does contribute to reduce the standard error of the estimate of the comparative effect size of Z to ENFO. The non-inferiority power with optimal sample allocation is about 7% higher than the even allocation strategy for all these three sample sizes. Utilizing the optimal allocation based on the existing network improves the power further. Hence, from even allocation without the existing network to optimal allocation with the existing network, the non-inferiority power increases in two ways. One is to borrow information from the indirect comparison; the other is to optimize sample assignment.

**Table 2:** Power of testing non-inferiority for different sample sizes under different sample allocation scenarios. Sample size is the total number of samples available in the new trial. $p_{NC}$ is the probability of retreatment after beef cattle being treated with negative control. ENFO is enrofloxacin; Z is the new treatment. Sample allocation is the number of samples assigned to each treatment. For example, (87, 1108, 1205) means the number of samples assigned to NC, ENFO, and Z are 87, 1108, and 1205

total sample size	$p_{NC}$	$p_{ENFO}$	$p_{Z}$	sample allocation (NC, ENFO, Z)	with existing network	non-inferiority power
2400	0.681	0.2229	0.2229	(800, 800, 800)	No	50.43%
2400	0.681	0.2229	0.2229	(800, 800, 800)	Yes	58.28%
2400	0.681	0.2229	0.2229	(87, 1108, 1205)	Yes	65.40%
3000	0.681	0.2229	0.2229	(1000, 1000, 1000)	No	58.33%
3000	0.681	0.2229	0.2229	(1000, 1000, 1000)	Yes	67.07%
3000	0.681	0.2229	0.2229	(87, 1408, 1505)	Yes	73.06%
3600	0.681	0.2229	0.2229	(1200, 1200, 1200)	No	65.17%
3600	0.681	0.2229	0.2229	(1200, 1200, 1200)	Yes	73.65%
3600	0.681	0.2229	0.2229	(87, 1708, 1805)	Yes	80.51%

#### Specific desired power

It is a common practice to specify a desired power of testing the research hypothesis in designing a new trial. Here we set the desired power to be 80% as an example for illustration purposes. The standard error needed to reach the 80% power is back calculated from Eq. 7 for the same non-inferiority scenario as in the previous subsection. It is then used to determine the needed total sample size based on Eq. 6 for analyses with the existing network and Eq. 2 for analyses without the existing network. Table 3 shows the total sample size, sample allocation and power obtained from the 10,000 simulated trials under this scenario. Using the existing network and uneven allocation enables a substantially smaller sample size which is 3559, about 40% less than the one without the existing network (5355).

**Table 3:** Calculated sample size needed under different sample allocation scenarios to reach 80% non-inferiority power.Sample size is the total number of samples available in the new trial. $p_{NC}$ is the probability of retreatment after beef cattle being treated with negative control. ENFO is enrofloxacin; Z is the new treatment. Sample allocation is the number of samples assigned to each treatment. For example, (87, 1687, 1785) means the number of samples assigned to NC, ENFO, and Z are 87, 1687, and 1785

total sample size	$p_{NC}$	$p_{ENFO}$	$p_{Z}$	sample allocation (NC, ENRO, Z)	allocation type	with existing network	non-inferiority power
5355	0.681	0.2229	0.2229	(1785, 1785, 1785)	even	No	80.01%
4584	0.681	0.2229	0.2229	(1528, 1528, 1528)	even	Yes	80.00%
3559	0.681	0.2229	0.2229	(87, 1687, 1785)	uneven	Yes	80.02%

### Trial scenario 2: testing superiority

Here we utilize the same BRD data to illustrate our proposed method for assessing superiority with an example new three-arm trial. The new three-arm trial involves NC, treatment Ceftiofur Sodium (CEFTS), and a new treatment of interest Z. The new treatment Z is supposed to have the same efficacy as CEFTS and the research question of interest is to detect a difference between the new treatment Z and NC. Here we set the new treatment to have the same efficacy as CEFTS instead of ENFO (which was used in previous illustrations) because the difference between CEFTS and NC is moderate so that it gives good example for illustrating the difference in testing power between methods. The powers of testing superiority based on simulation results for different sample sizes and under different sample allocation strategies are shown in Table 4. As with the previous example, the increase in power benefits from two aspects, the existing network and the optimized sample allocation. The increase in power, which is not fixed, is related to the total sample size, the log odds ratio of the treatments of interest and the existing network. For example, the power goes from 31% to 48% given the sample size is 60 while the difference in power is only 12% if the sample size is 180. The gain in power from the optimal sample allocation compared to the even allocation is about 3% in all three sample size scenarios. Similar to the non-inferiority example, the fixed power situation can also be applied in this superiority example to determine the total sample size to reach a certain power. Note that the sample allocation ratio of NC to Z changes dramatically as the sample size goes from 60 to 180. This is because in the superiority testing, NC is included in the comparison of interest. When the sample size is 60, the information from the indirect comparison NC-CEFTS-Z is more than that from the direct. Therefore, nearly all samples are allocated to CEFTS and Z. Because we set the minimum number of samples for each treatment to be ten in the optimal allocation strategy, there are still ten samples allocated to NC, which is used to rationalize the three-arm trial. As the sample size increases, information from the direct comparison becomes dominant hence the allocation optimization puts more weight on both treatments which are NC and Z in the comparison of interest.

**Table 4:** Power of testing superiority of Z to NC for different total sample sizes under different sample allocation scenarios. Sample size is the total number of samples available in the new trial. $p_{NC}$ is the probability of retreatment after beef cattle being treated with negative control. CEFTS is Ceftiofur Sodium; Z is the new treatment. Sample allocation shows the number of samples assigned to each treatment. For example, (30, 31, 59) means the number of samples assigned to NC, CEFTS, and Z are 30, 31, and 59

total sample size	$p_{NC}$	$p_{CEFTS}$	$p_{Z}$	sample allocation (NC, CEFTS, Z)	with existing network	superiority Z to NC
60	0.681	0.4303	0.4303	(20, 20, 20)	No	31.97%
60	0.681	0.4303	0.4303	(20, 20, 20)	Yes	45.37%
60	0.681	0.4303	0.4303	(10, 20, 30)	Yes	47.81%
120	0.681	0.4303	0.4303	(40, 40, 40)	No	64.48%
120	0.681	0.4303	0.4303	(40, 40, 40)	Yes	74.43%
120	0.681	0.4303	0.4303	(30, 31, 59)	Yes	78.13%
180	0.681	0.4303	0.4303	(60, 60, 60)	No	80.56%
180	0.681	0.4303	0.4303	(60, 60, 60)	Yes	89.54%
180	0.681	0.4303	0.4303	(61, 31, 88)	Yes	92.89%

## Discussion

In this paper, a sample allocation strategy is proposed to improve the power of a new trial by leveraging evidence from an existing network meta-analysis when the total sample size is fixed. The results clearly show that if a prior network is available it is feasible to reduce the required sample size or increase the power if the prior network of evidence is utilized. Given the importance of maximizing the value of prior research and efficiently using current resources, the approach we propose has the potential to help researchers as they design experiment [27]. Further, this approach to trial design is entirely consistent with ethical animal use guidelines to replacement, reduction and refinement, by reducing the number of animals required for animal research [28].

Network meta-analysis provides a quantitative framework to enlighten the design of new trials [6]. Several prior studies have discussed approaches to planning future studies based on the existing meta-analysis or network meta-analysis [2, 4, 6]. These papers focus on designing a new trial within the network framework which means the treatments in the new trial are already in the existing network. While in our work, the focus is on trials with a treatment arm which has not been included in the existing network. Additionally, our work considers leveraging evidence from the network meta-analysis while most of the literature in this topic addresses how the existing network provides guidance on new trials. Moreover, in this paper, we optimize sample allocation to improve the power of the comparison of interest in the new trial given the sample size fixed.

The goal of this paper is to improve the power of a new trial by borrowing information from an existing network. The extent of improvement depends on many aspects such as the sample size, the precision of estimates in the existing network, etc. If the prior network is small it is possible that the improvement in power is minimal compared with the even allocation scenario without the existing network. Additionally, it is possible that the optimal allocation is extremely unbalanced (e.g. one sample allocated to NC in row three of Table 4) since the goal is only to maximize the power while the extremely unbalanced allocation may not be viable in reality. In these cases, it is suggested the new trial designer balance the benefit and cost of using an optimal allocation strategy. Although we suspect there may be resistance to the use of such method, particularly if the sample allocation is very uneven (e.g. very few samples in one treatment), this method provides researchers with an option to improving the power of the new trial, especially for trials with limited number of samples available.

### Limitations

Same as all methodologies on NMA, our proposed method is also based on the general NMA assumptions, namely exchangeability, transitivity and consistency. The consistency assumption is a form of exchangeability and states that the direct and indirect evidence are in agreement. Such assumption may not always be plausible in practice. When heterogeneity is present, NMA analyses and results based on the consistency assumption are subject to bias. Assumption similar to the consistency has been studied in the context of supplementing new clinical trials with historical control data [29]. One important feature of such work is to discount historical information relative to information from the new trial, to avoid potential biases. It has been shown that there is difficulty in determining the appropriate degree of discounting the historical [30, 31]. Galwey [31] has also shown that conclusions can be highly sensitive to assumptions about differences between the historical and the new data.

### Future directions

Both fixed effects and random effects NMA models have been widely used in literature [1, 2, 5, 19], with the major difference being that the random effects NMA model contains a random effect of between study variation. This between study variation in the new treatment can not be assessed without NMA when there is only one new trial that contains the new treatment. We thus develop our proposed methodology in the framework of fixed effects NMA so that the scope of inference are comparable to the traditional standalone approach to analyzing the single new trial. It is possible to derive formulas in the framework of random effects NMA based on the same concepts as presented in this paper, which might be of interest to some researchers, especially when multiple new trials that contain the new treatment are being planned. Homogeneous between-study variance is a key assumption and needs to be assessed in constructing in random effects NMA models [32].

Methods exist for detecting and addressing the inconsistency in the context of NMA, e.g., the ‘inconsistency factors’ model, and the inconsistency degrees of freedom (ICDF) [33]. Van Rosmalen et al. [34] used simulation studies to show that methods estimating parameters for the between-trial heterogeneity generally offer the best trade-off between power, precision and type I error. It would be feasible to extend our proposed method to inconsistency NMA models that would accommodate and estimate the inconsistency parameters.

The proposed study can be extended to neutrosophic statistics [35, 36] which is the extension of classical statistics and is applied when the data is coming from a complex process or from an uncertain environment.

## Data Availability

We provide the R code and data we used in this paper in https://github.com/dapengh/Leveraging-evidence-from-NMA. The website address of the R shiny app is https://amoconnor.shinyapps.io/NMA-three-arms-sample-size/.

## Abbreviations

RCT: Randomized controlled trials
NMA: Network Meta-Analysis
FDA: Food and Drug Administration
BRD: Bovine Respiratory Disease
NC: Negative control
ENFO: Enrofloxacin
CEFTS: Ceftiofur Sodium
